# Advances in Research on Brain Health and Dementia: Prevention and Early Detection of Cognitive Decline and Dementia: Series II

**DOI:** 10.3390/brainsci16010108

**Published:** 2026-01-19

**Authors:** Takao Yamasaki

**Affiliations:** 1 Department of Neurology, Minkodo Minohara Hospital, Fukuoka 811-2402, Japan; yamasaki@kyudai.jp; Tel.: +81-92-947-0040; 2 Department of Research and Development, Kumagai Institute of Health Policy, Fukuoka 816-0812, Japan

## 1. Introduction

Dementia is a rapidly growing global health challenge, underscoring the urgent need for effective strategies aimed at the prevention and early detection of cognitive decline [1,2]. Research on brain health and dementia has advanced substantially in recent years, encompassing epidemiology, neuroimaging, cognitive neuroscience, and intervention science, as well as broader clinical, psychological, and technological disciplines. However, a critical challenge remains: translating these scientific advances into clinically meaningful insights and real-world applications that can effectively support individuals at risk and inform clinical practice.

Building on the conceptual framework established in our previous Special Issue (Series I) [3,4], this second series shifts its focus from defining brain health and dementia-related conditions to examining their translational and functional implications. Whereas Series I provided a comprehensive overview of epidemiological perspectives, diagnostic tools, biomarkers, and intervention strategies [3,4], Series II emphasizes how this growing body of evidence can be operationalized to support early detection, prevention, and everyday functioning in real-world and clinical contexts.

This Special Issue (Series II) brings together six original and review articles that collectively form a coherent and progressive narrative. The included studies address emerging societal and medical risk factors for cognitive vulnerability, subtle cognitive and functional changes observed at the mild cognitive impairment (MCI) stage, and clinically meaningful outcomes—such as decision-making capacity and everyday functioning—that directly affect autonomy and quality of life. Through this continuum, the contributions highlight early and potentially modifiable markers of cognitive decline and explore their implications for timely intervention.

## 2. Translational Perspectives on Brain Health

Building on the translational focus outlined in the Introduction, Section 2 presents the six contributions in a structured sequence, progressing from risk identification and early cognitive changes to functionally relevant outcomes and intervention-oriented approaches.

### 2.1. Emerging Societal and Medical Risk Factors for Cognitive Vulnerability

The COVID-19 pandemic has introduced a novel and increasingly important challenge to brain health research: the long-term cognitive and neurological sequelae collectively referred to as long COVID [5]. In this Special Issue, Rudroff (Contribution 1) provides a timely review positioning long COVID as an emerging societal and medical risk factor with direct relevance to cognitive decline and dementia research.

The review synthesizes evidence indicating that long COVID is frequently associated with persistent cognitive, neurological, and neuropsychiatric symptoms, even following mild acute infection. Importantly, Rudroff emphasizes that these post-infectious effects may confound findings in brain health studies if not systematically considered. This concern is particularly relevant for neuroimaging and brain stimulation research, where unrecognized long COVID–related alterations may overlap with or mimic early neurodegenerative changes.

From a translational perspective, this contribution underscores the need to adapt research designs to post-pandemic realities. Systematic screening for COVID-19 history, stratified analyses, and longitudinal follow-up are highlighted as essential steps to ensure valid interpretation of brain health data. By framing long COVID as both a confounding factor and a potential contributor to long-term cognitive vulnerability, this review sets the stage for integrating emerging societal risks into contemporary dementia prevention research.

### 2.2. Subtle Cognitive Changes at the Preclinical and MCI Stage

Beyond societal risk factors, identifying subtle brain and cognitive changes that precede overt impairment remains a central challenge in dementia prevention [6]. Lin et al. (Contribution 2) address this issue by examining associations among body mass index (BMI), hippocampal subfield volumes, and cognitive performance in non-demented older adults.

Their findings demonstrate that higher BMI is associated with structural alterations in a hippocampal subregion implicated in early medial temporal lobe vulnerability, particularly among older women. These neuroanatomical differences were accompanied by lower performance in specific cognitive domains, suggesting that metabolic factors may influence brain integrity and cognition well before clinical thresholds for MCI are reached.

This study provides important translational insights by highlighting BMI as a potentially modifiable risk factor with sex-specific effects on brain health. Moreover, the use of hippocampal subfield analysis illustrates how advanced neuroimaging approaches can detect early, functionally relevant markers that may not be captured by global measures alone. Such markers may be valuable for identifying individuals at heightened risk and for informing targeted, early-stage preventive interventions.

### 2.3. Functional Outcomes and Implications for Early Intervention

While early cognitive and neurobiological changes are critical, their clinical relevance ultimately depends on how they affect everyday functioning [7]. Alfeo et al. (Contribution 3) focus on this dimension by systematically reviewing decision-making capacity in individuals with MCI.

Across the reviewed studies, impaired decision-making in MCI emerged as a multifaceted phenomenon shaped by interactions among cognitive domains, emotional regulation, and daily functioning. Deficits in executive function and memory were consistently linked to poorer decision-making, while affective factors such as apathy and reduced insight further influenced real-world choices. Importantly, the review demonstrates that even subtle cognitive changes may translate into clinically meaningful impairments in everyday autonomy—particularly in financial and medical decision-making—prior to overt loss of independence.

By conceptualizing decision-making as an integrative functional outcome, this review highlights a key translational gap between standard cognitive assessments and everyday competence. Within the framework of Series II, this contribution reinforces the importance of incorporating functional and emotional dimensions into early assessment and intervention strategies, particularly when the goal is to preserve autonomy and quality of life.

### 2.4. Multimodal and Innovative Intervention Approaches

Moving from risk characterization and functional vulnerability toward actionable solutions, accumulating evidence suggests that effective prevention of cognitive decline and dementia requires multimodal and integrative intervention approaches [1,2]. Qian et al. (Contribution 4) demonstrate that both physical exercise and foreign language learning are associated with improvements in learning and selected cognitive domains in older adults. Their findings highlight the potential of lifestyle-based interventions and suggest that combining physical and cognitive engagement may offer complementary benefits, while also underscoring the importance of task design and intervention timing.

Extending this behavioral perspective, Maneemai et al. (Contribution 5) introduce sensory integration as a novel framework for healthy ageing and dementia management. By emphasizing the role of multisensory processing in cognition, emotion, and motor behavior, this review suggests that sensory-based interventions may offer functionally meaningful and personalized approaches to supporting brain health.

At the technological frontier, Stefanski et al. (Contribution 6) explore the integration of neurostimulation therapies with digital health platforms through cerebrovascular modeling approaches. Their review illustrates how systems-level and engineering perspectives can inform safer and more personalized neurostimulation strategies, thereby bridging basic neuroscience with precision-oriented clinical applications.

Collectively, these three contributions underscore the need for multidimensional intervention strategies that integrate lifestyle factors, sensory processes, and advanced technologies. Within Series II, they exemplify how translational brain health research can move beyond single-modality solutions toward scalable, integrative, and real-world interventions.

## 3. Conclusions

This Special Issue brings together six contributions that collectively illustrate how contemporary brain health research can be translated into functionally meaningful and clinically relevant insights. Rather than focusing solely on diagnostic classification, the studies included here emphasize early cognitive vulnerability, real-world functioning, and clinically meaningful endpoints that directly affect autonomy and quality of life.

Across diverse methodological and disciplinary perspectives, Series II underscores that effective prevention and early intervention will require integrative approaches that combine sensitive assessment, functional relevance, and multimodal intervention strategies. By aligning neuroscientific insight with real-world applicability, this Special Issue contributes to a more actionable and person-centered understanding of brain health across the ageing continuum.
